# Genetic and cellular sensitivity of *Caenorhabditis elegans* to the chemotherapeutic agent cisplatin

**DOI:** 10.1242/dmm.033506

**Published:** 2018-06-21

**Authors:** Francisco Javier García-Rodríguez, Carmen Martínez-Fernández, David Brena, Dmytro Kukhtar, Xènia Serrat, Ernest Nadal, Mike Boxem, Sebastian Honnen, Antonio Miranda-Vizuete, Alberto Villanueva, Julián Cerón

**Affiliations:** 1 Modeling human diseases in *C. elegans*. Genes, Diseases and Therapies Program, Bellvitge Biomedical Research Institute - IDIBELL, L'Hospitalet de Llobregat, 08908 Barcelona, Spain; 2 Program Against Cancer Therapeutic Resistance (ProCURE), Catalan Institute of Oncology (ICO), Bellvitge Institute for Biomedical Research (IDIBELL), L'Hospitalet del Llobregat, 08908 Barcelona, Spain; 3 Thoracic Oncology Unit, Department of Medical Oncology, Catalan Institute of Oncology (ICO), L'Hospitalet de Llobregat, 08908 Barcelona, Spain; 4 Division of Developmental Biology, Department of Biology, Faculty of Science, Utrecht University, 3584 CH Utrecht, The Netherlands; 5 Heinrich Heine University Düsseldorf, Medical Faculty, Institute of Toxicology, D-40225 Düsseldorf, Germany; 6 Instituto de Biomedicina de Sevilla, Hospital Universitario Virgen del Rocío/CSIC/Universidad de Sevilla, E-41013 Sevilla, Spain

**Keywords:** *Caenorhabditis elegans*, RNA-seq, Cisplatin

## Abstract

Cisplatin and derivatives are commonly used as chemotherapeutic agents. Although the cytotoxic action of cisplatin on cancer cells is very efficient, clinical oncologists need to deal with two major difficulties, namely the onset of resistance to the drug and the cytotoxic effect in patients. Here, we used *Caenorhabditis elegans* to investigate factors influencing the response to cisplatin in multicellular organisms. In this hermaphroditic model organism, we observed that sperm failure is a major cause of cisplatin-induced infertility. RNA sequencing data indicate that cisplatin triggers a systemic stress response, in which DAF-16/FOXO and SKN-1/NRF2, two conserved transcription factors, are key regulators. We determined that inhibition of the DNA damage-induced apoptotic pathway does not confer cisplatin protection to the animal. However, mutants for the pro-apoptotic BH3-only gene *ced-13* are sensitive to cisplatin, suggesting a protective role of the intrinsic apoptotic pathway. Finally, we demonstrated that our system can also be used to identify mutations providing resistance to cisplatin and therefore potential biomarkers of innate cisplatin-refractory patients. We show that mutants for the redox regulator *trxr-1*, ortholog of the mammalian thioredoxin reductase 1 *TRXR1*, display cisplatin resistance. By CRISPR/Cas9, we determined that such resistance relies on the presence of the single selenocysteine residue in TRXR-1.

This article has an associated First Person interview with the first author of the paper.

## Abstract (precis)

**Summary:** *Caenorhabditis* *elegans* is a valuable model to identify genetic factors influencing the animal response to the widely used chemotherapeutic agent cisplatin.

## INTRODUCTION

The US Food and Drug Administration approved the use of cisplatin [CDDP, cis-diammine-dichloroplatinum(II)] as a chemotherapeutic agent in 1978. Since then, cisplatin and other platinum-based derivatives have been used successfully in cancer treatment. To illustrate their impact in the clinic, it has been estimated that approximately half of all patients undergoing chemotherapeutic treatment receive a platinum drug (Galanski, 2006). Cisplatin exerts activity against a wide spectrum of solid neoplasms, including testicular, bladder, ovarian, head and neck, gastric and lung cancers (Dasari and Bernard Tchounwou, 2014). Strikingly, testicular cancer was previously fatal, but treatment with cisplatin provided a cure for 80% of the patients (Gonzalez-Exposito et al., 2016). Despite its effectiveness, there are patients intrinsically resistant to cisplatin-based therapies, and an important fraction of tumors eventually develop chemoresistance (Amable, 2016).

Cisplatin is composed of a double-charged platinum ion surrounded by four ligands, two amines and two chlorides. Inside cells, the low chloride concentration facilitates cisplatin aquation, replacing chloride groups by water molecules. This process produces a hydrolyzed (or aquated) form of cisplatin that is a potent electrophile (attracted to electrons) that can react with any nucleophile, including nucleic acids and the sulfhydryl groups of proteins (Kelland, 2007).

Cisplatin activity has an impact in the nucleus and in the cytoplasm. In the nucleus, cisplatin produces DNA intra- and interstrand crosslinks that lead to apoptosis (Kelland, 2007). In the cytoplasm, owing to its electrophilic activity, cisplatin behaves as an oxidant (loss of electrons results in oxidation), binding to proteins, including mitochondrial proteins, and especially to thiol groups (-SH). Thus, cisplatin produces a reactive oxygen species homeostasis imbalance that leads to more oxidizing conditions, which violate normal cellular function and, ultimately, can also promote apoptosis (Wang and Lippard, 2005). As a result of its broad and unspecific mode of action, cisplatin also affects normal cells. Nephrotoxicity, neurotoxicity and ototoxicity are some of the dose-limiting side effects reported upon cisplatin therapy (Kelland, 2007). Nevertheless, the major obstacle for the clinical efficacy of cisplatin as an anticancer drug is the chemoresistance developed by tumors rather than its toxicity in normal cells.

The acquisition of cisplatin resistance is multifactorial. The mechanisms by which tumor cells become resistant to the action of cisplatin have been classified into three types (Galluzzi et al., 2014): (1) pre-target mechanisms, i.e. reducing intracellular accumulation of cisplatin or increasing sequestration of cisplatin by nucleophilic scavengers as glutathione (GSH), metallothioneins and other cysteine-rich proteins; (2) on-target mechanisms, i.e. acquiring the ability to repair adducts or becoming tolerant to unrepaired DNA lesions; and (3) post-target mechanisms, i.e. hampering the execution of apoptosis in response to DNA damage (Siddik, 2003).

*Caenorhabditis* *elegans* is a well-established organism for the study of signaling pathways in response to drug exposure (Kaletta and Hengartner, 2006). Previous studies, performed in distinct biological contexts (Hemmingsson et al., 2010; Collis et al., 2006), have revealed the value of *C. elegans* to identify genes related to the cisplatin response (Table S1). Here, we show, for the first time, that cisplatin in *C. elegans* produces DNA adducts and a systemic response driven by two conserved transcription factors. We have uncovered a sperm-specific sensitivity to cisplatin and have demonstrated that resistance of the nematode to cisplatin relies on the presence of a single selenocystein of the thioredoxin reductase TRXR-1. Thus, this report is a more comprehensive study of the global response of *C. elegans* to cisplatin that also establishes a reliable methodology for future studies on mechanisms of resistance to cisplatin that would continue contributing to the search for new targets and markers for the benefit of cisplatin-based therapies.

## RESULTS

### A reliable assay to study the effect of cisplatin in *C. elegans*

**Fig. 1.**
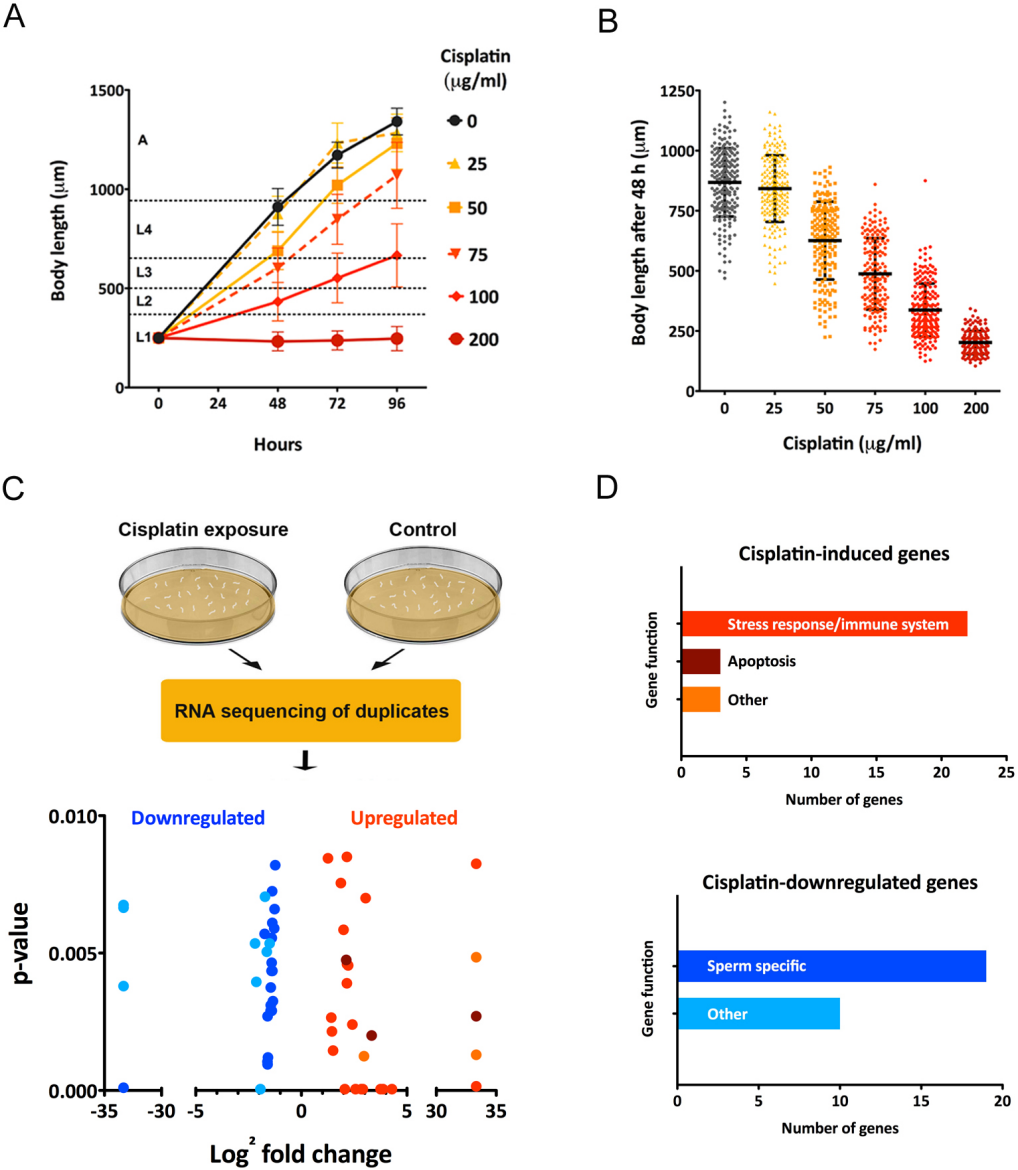
**Dose–response effect of cisplatin on *C. elegans* development and RNA-seq of animals exposed to cisplatin.** (A) Effect of distinct concentrations of cisplatin on larval development. Synchronized L1 larvae were grown on agar plates and exposed to different doses of cisplatin for 96 h at 20°C. Body length values represent mean and SD (*n*=50) of two different experiments. (B) Body length of nematodes that were grown from L1 stage exposed to different doses of cisplatin for 48 h at 20°C. Bars represent mean and SD of three independent replicates (*n*=50, *N*=3). (C) Schematic representation of transcriptomic analyses. Wild-type *C. elegans* from mixed stages were cultured with and without cisplatin (60 µg/ml) for 24 h at 20°C. Then, total mRNA of two biological replicates was purified and sequenced. Volcano plot represents genes significantly up- and downregulated (*P<*0.01) in cisplatin-treated versus untreated animals. (D) Color code represents the functional categories of up- or downregulated genes. Genes were clustered according to the gene functional classification tool DAVID and GO terms retrieved from WormBase.

In *C. elegans* nematodes, cisplatin produces a wide variety of phenotypes depending on the concentration, length of treatment and developmental stage of the treated animals (Tables S1 and S2). To implement a reliable methodology and systematically investigate the response of *C. elegans* to cisplatin, we established dose–response patterns during larval development, when cell divisions occur in somatic and germ cells. We exposed a synchronized population of L1 larvae on nematode growth medium (NGM) plates to different cisplatin concentrations for 96 h. We observed effects ranging from a developmental delay at 50 µg/ml to a larval arrest at 200 µg/ml (Fig. 1A). Based on this assay, we concluded that body length at 48 h post-L1, upon cisplatin exposure from 50 to 75 µg/ml, is a reliable indicator of the effect of cisplatin during *C. elegans* development (Fig. 1B). Thus, we established a methodology to investigate how distinct treatments or gene activities can influence the response of *C. elegans* to cisplatin.

### Transcriptional response of *C. elegans* to cisplatin

To explore the global response of nematodes to cisplatin, we studied transcriptional signatures in RNA sequencing (RNA-seq) experiments. We performed this new transcriptome analysis using a mixed-stage nematode population that was treated for 24 h with 60 µg/ml of cisplatin, because this dosage of cisplatin produces some phenotypes, but does not compromise the viability of the animals. To reduce the number of false positives, we used two biological replicates to compare the transcriptomes of treated and non-treated populations (Fig. 1C). We used the Cufflinks algorithm (Trapnell et al., 2012) to process the RNA sequencing data and study differential expression of genes. We identified a set of 83 genes upregulated and 78 genes downregulated by cisplatin in both experiments (*P*≤0.05; Table S3). A more restricted list of candidates, using a cut-off *P*-value of ≤0.01, included 28 genes upregulated and 29 downregulated upon cisplatin exposure.

To explore the functions of these 57 genes, we performed a gene ontology (GO) analysis and identified predicted protein domains (COG) (Tatusov et al., 2003; Huang et al., 2009) (Fig. 1D). Genes encoding CUB-like domains, C-type lectins and glutathione-*S*-transferases were found among the genes upregulated upon cisplatin exposure. In *C. elegans*, the expression of such genes is associated with detoxification, redox balance, stress response and innate immune system, and it is often regulated by the transcription factors DAF-16 and SKN-1 (Singh and Aballay, 2009; Park et al., 2009; Yen et al., 2011; Tepper et al., 2013) (Table S4). We also detected an overlap with genes induced upon ionizing radiation (IR) (Greiss et al., 2008; Schumacher et al., 2005a,b) (Table S4). Among those, we found genes involved in the apoptotic signaling cascade, including *egl-1* and *ced-13* (Greiss et al., 2008). However, as described below, these two genes function in a distinct manner upon cisplatin exposure.

Of the 29 genes downregulated by cisplatin, we found that most of those were sperm-specific genes (Ortiz et al., 2014), with the Major Sperm Protein (MSP) domain as the most frequent GO annotation (Table S5). Therefore, the reduced brood size induced by cisplatin might be provoked, at least in part, by a specific effect of this agent on spermatogenesis and/or on sperm activity.

### Cisplatin reduces the germ cell pool and affects sperm functionality

**Fig. 2.**
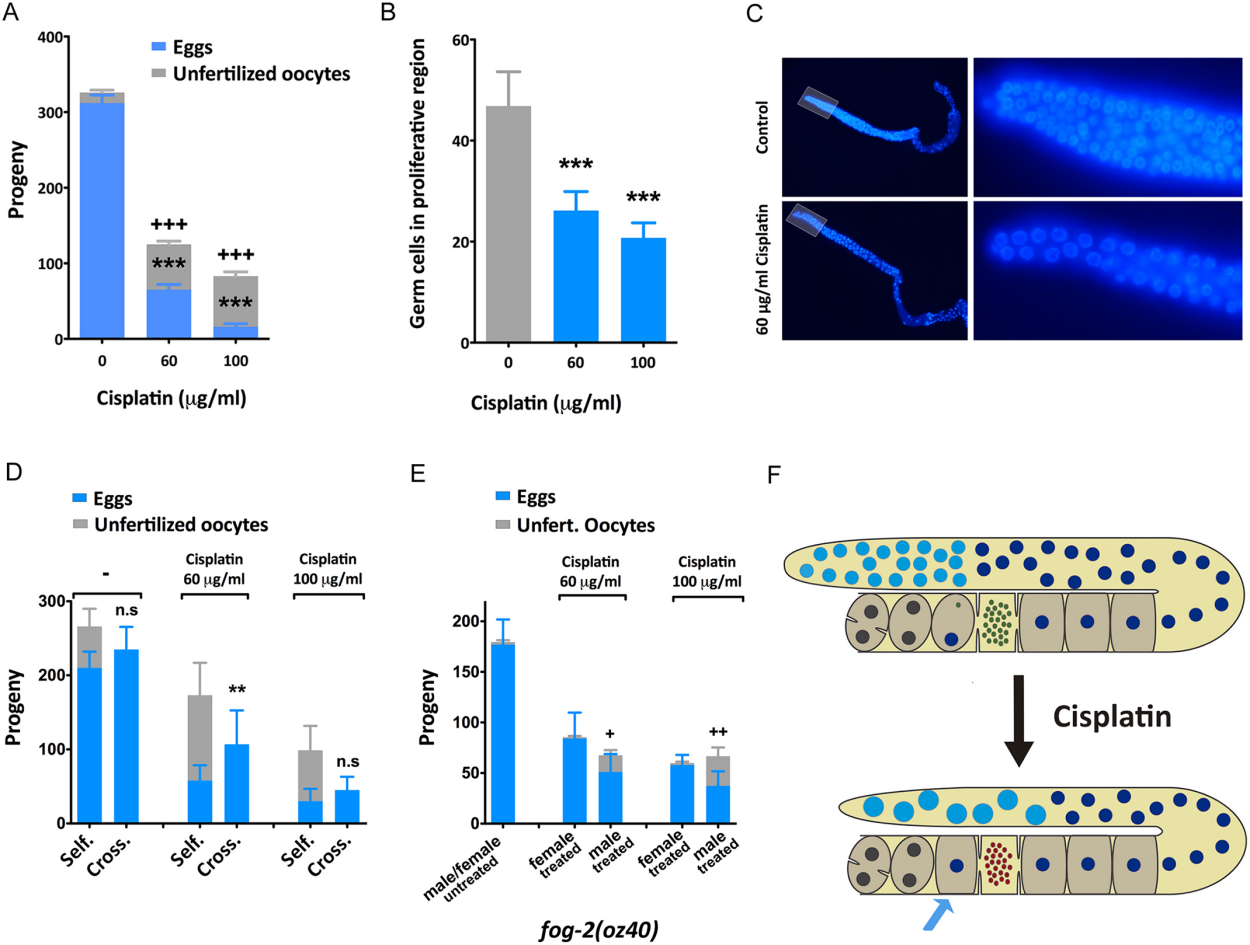
**Effect of cisplatin in the germline.** (A) Cisplatin reduces brood size and increases the percentage of unfertilized oocytes laid. L4 animals were exposed to cisplatin for 24 h at the indicated concentrations (*n*=12, *N*=2). (B) Cisplatin reduces the number of germ cells in the proliferative region of *C. elegans* germline. Number of cells corresponds to nuclei observed, in a single *z*-stack, at the proliferative region (50 μm away from the distal end of the gonad) (*n*=15, *N*=2). (C) Representative DAPI staining of young adult germline exposed to cisplatin for 24 h. Right panels show magnification of the germline proliferative region (highlighted area). (D) Impact of cisplatin on fertilization. Cisplatin produces unfertilized oocytes and causes reduced brood size in self-fertilized hermaphrodite worms (‘Self’), but this effect is rescued, in part, by crossing cisplatin-treated hermaphrodites with untreated males (‘Cross’; *n*=12, *N*=2). ***P>0.001, **P>0.01, *P>0.1; n.s., non-significant. (E) Cisplatin-induced unfertilized oocytes are caused by defective sperm. *fog-2(oz40)* hermaphrodites do not produce sperm, and mating is necessary to maintain the strain. Cisplatin produces unfertilized oocytes only when males are treated (*n*=12, *N*=2). ^++^*P*>0.01, ^+^*P*>0.1 compared with female treatment in the same conditions. (F) Schematic diagram to illustrate the effect of cisplatin in the germline: bigger nuclei at the proliferative zone (light blue), defective sperm (red dots) and unfertilized oocytes (blue arrow). Bars show mean and s.e.m., and Student's *t*-test was applied.

Our transcriptome analyses indicated that male germline genes are particularly downregulated in the presence of cisplatin. To investigate the effect of cisplatin on the germline, we exposed L4 animals to cisplatin for 24 h, because the switch from spermatogenesis to oogenesis occurs at this stage and therefore both processes can be affected (Ellis and Schedl, 2007). As expected, we observed a dose-dependent reduction of the brood size upon cisplatin exposure (Meier et al., 2014) (Fig. 2A). This reduction was correlated with an increase in the number of unfertilized eggs laid (Fig. 2A) and a decrease in the number of cells at the proliferative region of the germline (Fig. 2B). We observed that cisplatin-treated germlines displayed fewer nuclei in the mitotic region that appeared to be bigger (Fig. 2C), which is an effect described in germlines exposed to DNA-damaging agents that is attributable to cell cycle arrest upon activation of the S-phase checkpoint (Gartner et al., 2004).

The increased number of unfertilized oocytes is a phenotypic hallmark of sperm failure in *C. elegans* (Kadandale and Singson, 2004). To investigate whether the unfertilized oocytes observed upon cisplatin treatment were the result of defective sperm, we crossed cisplatin-treated hermaphrodites with untreated males. We observed that sperm from untreated males rescued, at least in part, the brood size and abrogated the presence of unfertilized oocytes (Fig. 2D). These results suggest that the excess of unfertilized oocytes is attributable to the effect of cisplatin in spermatogenesis rather than in oogenesis.

To investigate the effect of cisplatin on sperm functionality, we used the female/male strain *fog-2(oz40)*, allowing the treatment of males and females independently before the cross. *fog-2(oz40)* females exposed to cisplatin and crossed with *fog-2(oz40)* untreated males showed a reduced brood size, as expected from the effect of cisplatin on mitotic cells, but we did not detect an increased production of unfertilized oocytes (Fig. 2E). On the contrary, by crossing *fog-2(oz40)* males exposed to cisplatin with untreated *fog-2(oz40)* females we observed a stronger reduction of the progeny, but also a higher number of unfertilized oocytes. These results confirm that, at a similar dose, cisplatin does not hamper the capacity of oocytes to be fertilized but affects the capability of sperm to fertilize.

In summary, the exposure to cisplatin produces smaller germlines as consequence of a reduction of the proliferative region and fertilization problems caused by the effect of cisplatin in the sperm (Fig. 2F).

### DAF-16 and SKN-1 are key players in the response to cisplatin

**Fig. 3.**
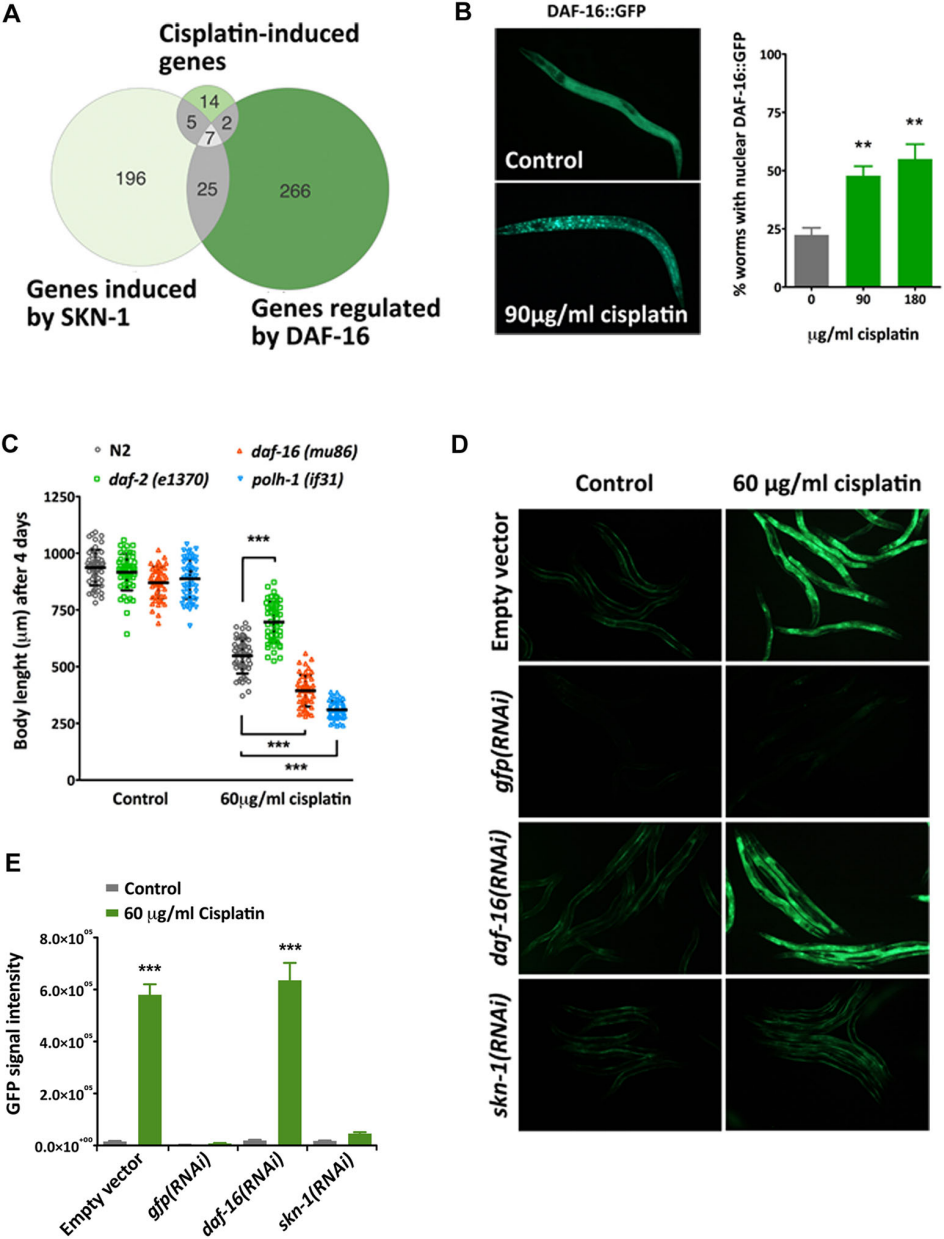
**Role of DAF-16/FOXO and SKN-1/Nrf2 transcription factors in the response to cisplatin.** (A) Venn diagrams showing the significant overlap between the genes induced by cisplatin found in our transcriptomic analyses and genes regulated by DAF-16 (Tepper et al., 2013) and SKN-1 (Oliveira et al., 2009). *P*<0.001 by Fisher's test. (B) Cisplatin induces DAF-16 nuclear translocation. Representative image of L2 larvae carrying a translational GFP reporter of DAF-16 with and without cisplatin (90 µg/ml for 5 h). The graph represents the percentage of synchronized L2 larvae with DAF-16::GFP predominantly nuclear after 5 h of exposure to cisplatin at the indicated concentrations. Bars represent mean and s.e.m. (*n*=50, *N*=3). **P<0.01 relative to untreated worms by Student's *t*-test. (C) IIS pathway influences the response to cisplatin. Body length of synchronized L1 larvae grown for 4 days in the absence or presence of cisplatin. Worms were grown at 15°C to avoid *daf-2(e1370)* dauer phenotype. The inactivation of the IIS pathway in *daf-2(e1370)* mutants, which keeps DAF-16 constitutively in the nucleus, causes resistance to cisplatin, whereas the *daf-16(mu86)* null mutant increases cisplatin sensitivity. Translesion synthesis polymerase 1 mutant allele *polh-1(if31)* was used as a positive control (Roerink et al., 2012). Bars represent mean and SD (*n*=50). ***P<0.001 relative to untreated worms by Student's *t*-test. (D) Cisplatin-induced activation of *gst-4* is regulated by SKN-1. Representative images of synchronized *gst-4*p::GFP L4/young adult animals grown at 20°C on *daf-16(RNAi)* or *skn-1(RNAi)* bacteria from the L1 stage in the presence or absence of cisplatin. *skn-1(RNAi)* inhibits the cisplatin-induced *gst-4* expression. Worms fed with *gfp* RNAi were used as a positive control. (E) Fluorescence intensity for each condition. Bars represent the mean. Error bars indicate the s.e.m. (*n*=30, *N*=2). ***P<0.001 relative to untreated animals by Student's *t*-test.

The abundance of dod (downstream-of-daf-16) genes and other stress response genes in the list of genes upregulated upon cisplatin exposure led us to study the role of DAF-16 and SKN-1. These are the orthologs of human FOXO3 and NRF2, respectively, and are required for stress resistance in *C. elegans* (Rodriguez et al., 2013)*.* Strikingly, we found that half of the upregulated genes were previously reported as DAF-16 and/or SKN-1 targets (Fig. 3A).

The evolutionarily conserved insulin/insulin-like growth factor signaling (IIS) pathway, through its main transcription factor DAF-16/FOXO, controls many different biological processes and regulates a wide variety of stresses, including starvation, oxidative stress (Honda and Honda, 1999), heavy metal toxicity (Barsyte et al., 2001) and ultraviolet radiation (Murakami and Johnson, 1996). DAF-16 is constitutively expressed and sequestered in the cytoplasm in its phosphorylated form. If the inhibitory phosphorylation is compromised, e.g. by reduced signaling of the DAF-2 receptor tyrosine kinase or stress conditions, DAF-16 will translocate to the nucleus. Using a DAF-16::GFP reporter strain, we observed that DAF-16 nuclear location increased upon cisplatin exposure in a dose-dependent manner (Fig. 3B), confirming the implication of the IIS pathway in the response to cisplatin.

Next, we investigated the extent to which the manipulation of the IIS activity could modify the response of the organism to cisplatin. We observed that *daf-2(e1370)* mutants, which are animals lacking a functional DAF-2/IGF-1-like receptor that constitutively induce DAF-16 nuclear localization (Yen et al., 2011), display resistance to cisplatin during larval development (Fig. 3C). Consistently, *daf-16(mu86)* mutants were hypersensitive to cisplatin (Fig. 3C), highlighting the relevance of DAF-16 nuclear activity in the cellular response to cisplatin.

Differently from *daf-2* and *daf-16*, *skn-1* is an essential gene hampering the use of loss-of-function mutants. To confirm the implication of SKN-1 in the response to cisplatin, we used a GFP reporter for one of its canonical targets, *gst-4*. SKN-1 regulates the stress-induced *gst-4* transcription in the presence of redox active compounds, such as paraquat or heavy metals (Tawe et al., 1998; Roh et al., 2006). In our RNA-seq data, *gst-4* is one of the SKN-1-regulated genes strongly induced by cisplatin, and we validated this result using a *gst-4* transcriptional GFP reporter (Fig. 3D,E). Moreover, using *skn-1* RNA interference (RNAi) we confirmed that the cisplatin induction of *gst-4* expression was *skn-1* dependent and *daf-16* independent (Fig. 3D,E). Thus, by studying the impact of cisplatin in the global gene expression we uncovered a systemic response of the organism that is driven by two conserved transcription factors. The fact that these transcription factors are effectors of metabolic and environmental signals opens new avenues to regulate the response to cisplatin in multicellular organisms.

### The BH3-only protein CED-13 protects against cisplatin

**Fig. 4.**
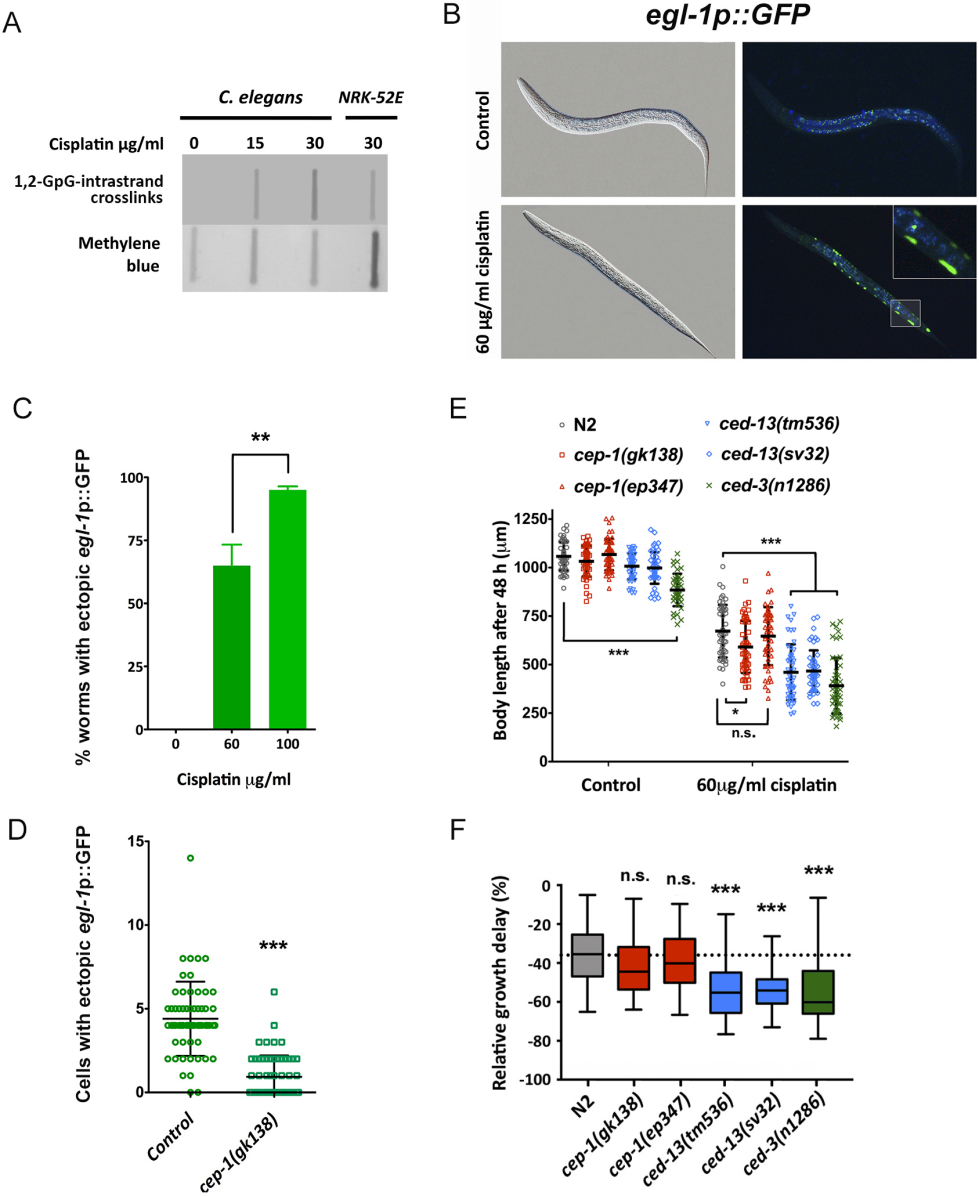
**Role of apoptotic pathways in the response of *C. elegans* to cisplatin.** (A) Southern blot showing that 1,2-GpG-intrastrand crosslinks are raised upon cisplatin exposure of a *C. elegans* population. There is a dose-dependent accumulation of DNA adducts in the presence of cisplatin. NRK-52E rat cells, in which 1,2-GpG-intrastrand crosslinks are not present (Krüger et al., 2016), were incubated with cisplatin as a positive control. (B) Cisplatin induces *egl-1* ectopic expression in somatic tissues during larval development. Representative images of L2 worms carrying a *egl-1*p::GFP reporter, untreated and treated with cisplatin (60 µg/ml for 24 h at 20°C). (C) Quantification of worms with ectopic *egl-1*p::GFP fluorescence in somatic cells of synchronized L1 larvae grown at the indicated concentrations of cisplatin for 24 h at 20°C. Bars represent mean and s.e.m. of three different experiments (*n*=50). **P<0.01 by Student's *t*-test. (D) *egl-1* ectopic induction in somatic cells requires *cep-1*/p53. Quantification of cells with ectopic *egl-1*p::GFP fluorescence of L1 larvae exposed to 60 µg/ml of cisplatin for 24 h at 20°C in wild-type and *cep-1(gk138)* mutants. Bars represent mean and s.e.m. of three different experiments (*n*=50). ***P<0.01 by Student's *t*-test. (E,F) Inactivation of the *cep-1*/p53 apoptotic pathway does not increase cisplatin resistance. (E) Body length of L1 larvae of wild-type and distinct mutant alleles grown in cisplatin (60 µg/ml) for 48 h at 20°C. Bars represent mean and s.d. (*n*=50). This experiment was performed three times with similar results. (F) Relative values of growth delay were obtained by calculating the percentage of the difference in the body length before and after cisplatin exposure (non-parametric Kruskal–Wallis test, ***P<0.001; *P< 0.05; n.s., non-significant).

We demonstrate, for the first time, that cisplatin leads to the formation of DNA adducts in *C. elegans* (Fig. 4A) and induces the expression of two apoptosis-related genes, *egl-1* and *ced-13*, which encode BH3-only proteins, which are a subset of the Bcl-2 familiy that contain only a BH3 domain and promote apoptosis. *egl-1* and *ced-13* are transcriptionally induced upon DNA damage (also upon exposure to UV or IR; Stergiou et al., 2007), and this induction is CEP-1*/*P53 dependent (Greiss et al., 2008). *Y47G7B.2*, another cisplatin-induced gene in our RNA-seq, encodes a nematode-specific gene that is also upregulated upon DNA damage in a *cep-1*-dependent manner (Greiss et al., 2008). Thus, the effects of cisplatin on upregulation of apoptotic genes could be attributable to *cep-1*-dependent DNA damage.

*egl-1* is required for DNA damage-induced apoptosis in both somatic tissues and the germline (Gartner et al., 2000). We investigated *egl-1* induction upon cisplatin exposure in the soma using a GFP reporter strain (*egl-1*p::GFP). During larval development, only 18 somatic cells of wild-type nematodes undergo apoptosis at the early L2 stage (Lettre and Hengartner, 2006). Strikingly, a 24 h cisplatin treatment in *C. elegans* (from L1 to L2) induced ectopic *egl-1*p::GFP expression that was evident in somatic cells (Fig. 4B,C). Moreover, this cisplatin-induced *egl-1* ectopic expression was, at least in part, *cep-1* dependent (Fig. 4D).

In the canonical DNA damage-induced apoptotic pathway, *cep-1* is upstream of the BH3-only proteins EGL-1 and CED-13. However, *ced-13*, and its downstream gene *ced-3*, have an additional role in the intrinsic apoptotic pathway that promotes protective changes rather than killing damaged irreparable cells (Yee et al., 2014). Accordingly, we found that *ced-13* and *ced-3* mutants were sensitive to cisplatin, whereas two different *cep-1* mutants were not resistant to cisplatin (Fig. 4E,F).

In summary, during *C. elegans* larval development, cisplatin induces the expression of *egl-1* and *ced-13*, but their influence is distinct. Inhibition of the canonical DNA damage-induced apoptotic pathway by using *cep-1* mutants does not produce animals resistant to cisplatin. On the contrary, *ced-13* and *ced-3* mutants are sensitive to cisplatin, underscoring a protective role of the intrinsic apoptotic pathway upon cisplatin exposure in somatic cells.

### TRXR-1 activity confers systemic sensitivity to cisplatin

The majority of *C. elegans* genes related to cisplatin response published to date present cisplatin sensitivity if inactivated by RNAi or mutation (Table S1). The human orthologs of these genes are potential targets of therapies to re-sensitize cisplatin-resistant tumors. Given the clinical importance of identifying refractory patients, we wondered whether our system was also valuable for the study of genes whose inactivation confers resistance to cisplatin. In the clinic, mutations or single nucleotide polymorphisms in these genes could function as predictive markers of cisplatin response. In human cell lines, thioredoxin reductase 1 (TRXR1, also known as TXNRD1) selenocysteine amino acid is a direct target of cisplatin, producing cytotoxic and highly pro-oxidant TRXR1 forms called SecTRAPs, which lead to high redox stress resulting in cell death (Anestål et al., 2008). TRXR1 is one of the few selenoproteins [proteins that include a selenocysteine (SeCys) amino acid] in mammals, but TRXR-1 is the only selenoprotein in *C. elegans* (Li et al., 2012) and, in contrast to mammals, this protein is not essential for viability (Stenvall et al., 2011). Depletion of *C. elegans trxr-1* does not produce phenotypes in terms of morphology, growth, lifespan, brood size or response to oxidative stress. It is essential only for larval molting when the glutathione reductase gene *gsr-1* is inactivated in parallel (Stenvall et al., 2011). All these features make *C. elegans* an excellent multicellular system in which to study specifically the role of the SeCys residue in the response to cisplatin.

**Fig. 5.**
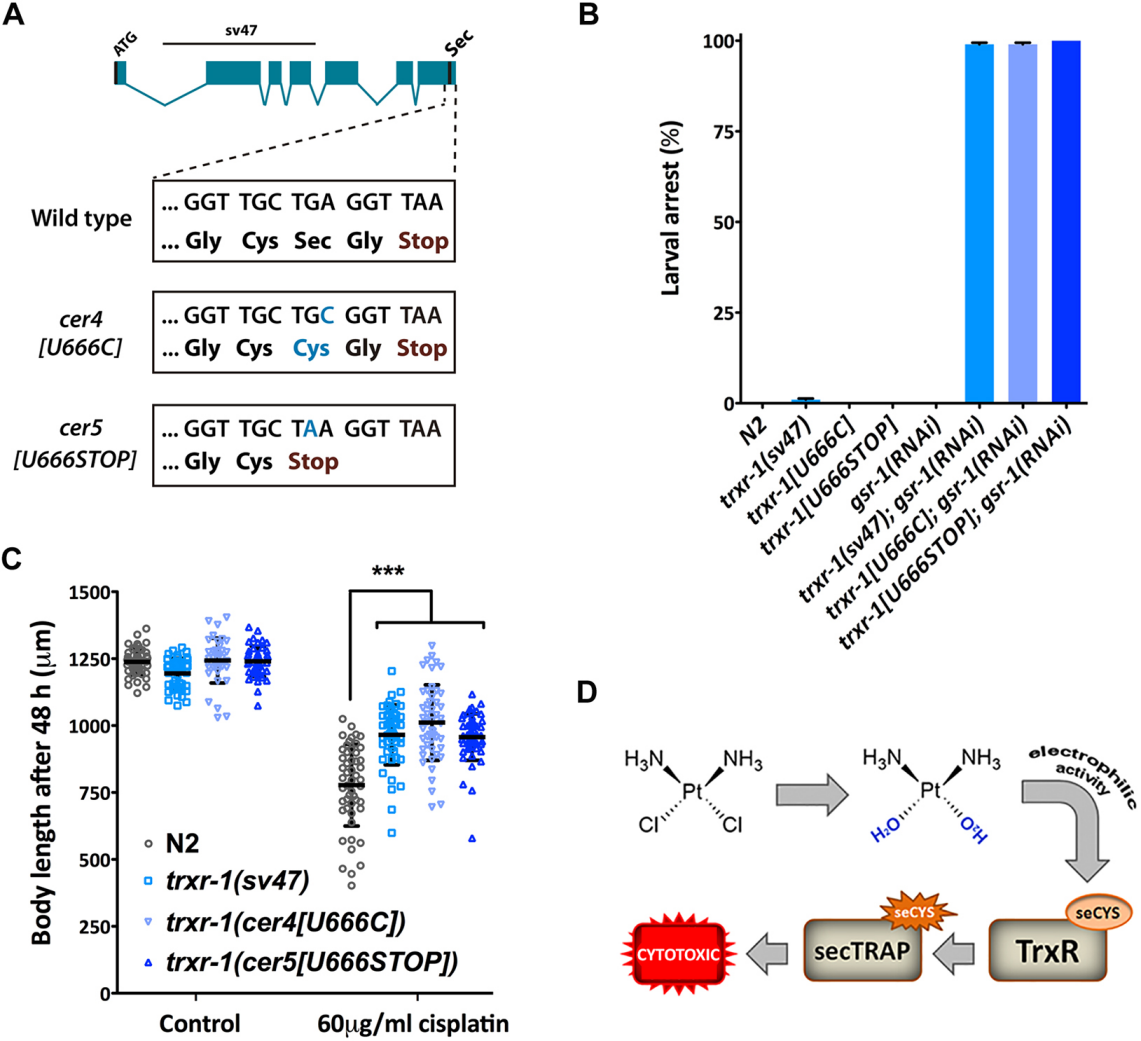
***trxr-1* null and missense mutants are resistant to cisplatin.** (A) Representation of the *trxr-1* gene. Blue boxes represent exons, and lines represent introns. The upper line denotes the 1663 bp region deleted in the *sv47* allele. Selenocysteine codon TGA is the third last residue and is represented as Sec. The wild-type sequence of the last four codons and their corresponding amino acids are boxed below. *cer4[U666C]* and *cer5[U666STOP]* Sec-deficient *trxr-1* mutant alleles, edited by CRISPR/Cas9, carry a single substitution in Sec codon (C instead of A or A instead of G) that changes the selenocysteine to cysteine or a premature STOP codon, respectively. (B) Sec-deficient *cer4*[U666C] and *cer5*[U666STOP] alleles, in a similar manner to the *trxr-1* null allele, cause a molting-associated growth arrest when fed with *gsr-1(RNAi).* Graph shows the quantification of larval arrest of L1 larvae fed with *gsr-1(RNAi)* or control bacteria (empty vector). Bars represent mean and s.d. (*n*=50, *N*=2). (C) *trxr-1(sv47)* null mutant and *cer4*[U666C] and *cer5*[U666STOP] point mutant strains show increased resistance to cisplatin. Body length quantification of synchronized L1 larvae grown on agar plates containing 0 and 60 µg/ml of cisplatin for 48 h at 20°C. Bars represent mean and s.d. (*n*=50). This experiment was performed in triplicate with similar results. ***P<0.001 relative to wild-type worms in the same conditions by Student's *t*-test. (D) Schematic diagram of the cytotoxic effect produced by the electrophilic activity of cisplatin when reacting with a selonocystein present in thioredoxin reductase 1.

We tested the robustness of our model by studying the role of thioredoxin reductase 1 (*trxr-1*) in the *C. elegans* response to cisplatin. We used the CRISPR/Cas9 system to produce two endogenous TRXR-1 variants, *trxr-1(cer4*[U666C]*)* and *trxr-1(cer5*[U666STOP]*)*, that change the selenocysteine for cysteine and a STOP codon, respectively (Fig. 5A). In a similar manner to the null mutant *trxr-1(sv47)*, these missense mutations showed larval arrest, attributable to molting defects, in combination with *gsr-1(RNAi)* (Fig. 5B). Strikingly, both the null mutant and the point mutations without the selenocysteine produce resistance to cisplatin (Fig. 5C). Thus, both the developmental function and the capacity to promote cytotoxicity in the presence of cisplatin rely on the presence of the selenocysteine residue.

Therefore, the electrophilic activity of hydrolyzed cisplatin in the presence of selenocysteine might produce cytotoxic SecTRAP forms that are major drivers of the harmful effect of cisplatin on multicellular organisms (Fig. 5D).

We also explored the role of *trxr-1* in sensitizing the animal to cisplatin. We found that *trxr-1(sv47)* confers resistance in germ cells and in somatic cells. Interestingly, by combining *trxr-1(sv47)* with *daf-2* and *daf-16* mutations, the IIS pathway is epistatic to the *trxr-1* activity on cisplatin resistance (Fig. S1).

## DISCUSSION

Despite three decades of clinical use and intense research, greater knowledge is necessary to understand, and ultimately control, the molecular events driving endogenous and acquired resistance to platinum-based chemotherapy (Dilruba and Kalayda, 2016). In this context, model organisms are platforms with growing interest in the study of the molecular, cellular and systemic responses to chemotherapeutic agents, but also to screen for new treatments (Nijman, 2015). Here, we reinforce the use of *C. elegans* to study the complex response of multicellular organisms to cisplatin. The function of *C. elegans* genes in the response to cisplatin has been shown in different biological contexts as adult survival or germline apoptosis (Table S1). After testing some of these contexts, we selected the body length of developmental larvae as a reliable and scalable system to evaluate the response of the animal to cisplatin. Nematodes are easy to synchronize at the L1 stage, and therefore we can study homogeneous populations during larval development, when somatic and germ cells are in active proliferation and apoptotic pathways functional.

The *C. elegans* germline is diverse in cell types, because it contains mitotic cells, meiotic cells, mature oocytes and sperm. We observed that the effect of cisplatin on fertility and cell cycle arrest in the mitotic germline is similar to that produced by other DNA-damaging insults, such as ionizing or ultraviolet C radiations (Gartner et al., 2004), suggesting a direct action of cisplatin on DNA that is supported by our southwestern blots for DNA adducts (1,2-GpG-intrastrand crosslinks). This cisplatin-induced DNA damage response (DDR) in the *C. elegans* germline has already been documented (van Haaften et al., 2006; Meier et al., 2014; Honnen, 2017). In addition, however, we found that defective sperm is a major cause of the reduced *C. elegans* fertility observed upon cisplatin exposure at L4. At this stage, we hit oogenesis only partially, but our experiments with *fog-2* mutants suggest that sperm and oocytes are different in terms of resistance to cisplatin. Interestingly, cisplatin is particularly efficient for testicular tumors and causes a drastic effect on spermatogenesis and sperm in treated patients, but this harmful effect seems to affect primarily the chromatin and is reversible (Bujan et al., 2013). In females with ovarian cancer, cisplatin also affects gametogenesis, producing a reduction of the ovarian reserve (Chang et al., 2015). Detailed experiments need to be performed to gain a better understanding of the step at which gametogenesis is hampered in the presence of cisplatin. In this sense, the hermaphrodite condition of *C. elegans* is a clear advantage to study the effect of any given cisplatin dose on oogenesis and spermatogenesis in the same organism.

Our RNA-seq analyses revealed a transcriptomic signature in the presence of cisplatin. The cisplatin-responsive genes identified could be involved in cell autonomous mechanisms (cells have an individual response to cisplatin uptake) or in non-cell autonomous mechanisms (the effect in certain cells influences distant cells). Given that DNA is damaged by cisplatin, a cell-autonomous response occurs as a checkpoint mechanism to favor DNA repair (Gartner et al., 2004). Nevertheless, the vast majority of genes induced by cisplatin in our experiments are related to general stress responses and the innate immune system, which suggests a coordinated systemic response. According to the deregulated genes, this response has DAF-16 and SKN-1 as major drivers and has similarities to that obtained from nematodes exposed to IR or UV-C (Boyd et al., 2010; Greiss et al., 2008). DAF-16 activation positively regulates a wider spectrum of processes, such as stress resistance, innate immunity and metabolic adaptation (Singh and Aballay, 2009; Murphy and Hu, 2013), whereas the NRF2 *C. elegans* ortholog SKN-1 is an important factor in detoxification and response to oxidative stress, and its downregulation has been associated with hypersensitivity to different stressor agents (Blackwell et al., 2015).

Interestingly, immune and stress reactions upon DNA damage are common in mammals (Ermolaeva and Schumacher, 2013), and similar to what we observed in *C. elegans*, cisplatin induces the nuclear translocation of the DAF-16 homolog FOXO3 (Fernandez de Mattos et al., 2008). Human FOXO proteins are crucial regulators of a multitude of cellular functions (Yang and Hung, 2009), but our results encourage further exploration of the manipulation of the IIS pathway to control the cellular response to cisplatin.

The SKN-1 human ortholog NRF2 regulates the expression of stress-responsive genes, such as SOD and catalases, or phase II detoxification enzymes, such as glutathione transferases (Kaspar et al., 2009). NRF2 overexpression or hyperactivation provokes a direct effect in acquisition of resistance to a wide spectrum of anticancer drugs in many cancer types (Gañán-Gómez et al., 2013; Kaspar et al., 2009). Accordingly, increased nuclear NRF2 expression has been shown in cisplatin-resistant human bladder cancer samples (Hayden et al., 2014). On the contrary, inhibition of NRF2 sensitizes cisplatin-resistant A549 cells (Hou et al., 2015). These observations are concordant with our findings in *C. elegans*, suggesting that direct or indirect targeting of NRF2 should be explored for cisplatin-combined therapeutic interventions.

Our transcriptomic analyses uncovered the upregulation of *egl-1* and *ced-13*, which are related to apoptosis. Using a *egl-1*p::GFP reporter, we found ectopic expression in somatic cells. The apoptotic pathway in postembryonic somatic cells is not well described and might be different in each somatic cell type. In the canonical view of the DNA damage-induced apoptotic pathway, expression of the effector EGL-1 marks cells destined to die (Nehme and Conradt, 2008), and *cep-1*/p53 is upstream (Schumacher et al., 2005a). However, *egl-1*p::GFP is still overexpressed in *cep-1*/p53 mutants exposed to cisplatin, indicating that inhibition of the DNA damage-induced apoptotic pathway does not completely protect the animal from cisplatin toxicity. Likewise, blockade of apoptosis in adult worms through *cep-1*/p53 inhibition does not have any effect on cisplatin sensitivity (Hemmingsson et al., 2010). Thus, inhibition of the DNA damage-induced apoptotic pathway is not an efficient strategy for animals to hamper the global effect of cisplatin. Nonetheless, expression of *cep-1*/p53 seems to be important for the full response to cisplatin, and p53 is required for the cytotoxic effect of cisplatin in human glioblastoma cells (Park et al., 2006).

Surprisingly, we found that *ced-13* upregulation might have a protective role, because *ced-13* mutants are sensitive to cisplatin. Mutants of *ced-3*, a gene that is genetically downstream, are also sensitive to cisplatin. This result fits with the role of *ced-13* in the mitochondrial intrinsic apoptotic pathway that extends *C. elegans* longevity (Yee et al., 2014). It is possible that postmitotic cells, which are irreplaceable, activate *ced-13* activity to protect themselves from cisplatin. The role of *ced-13* might be dependent on the insult levels or vary in distinct cell types, because overexpression of *ced-13* in the soma induces cell death of somatic cells that normally survive (Schumacher et al., 2005a). In concordance with a protective role for *ced-13* and *ced-3*, the activity of these genes appears to protect dopaminergic neurons from the toxicity of an oxidative stress-inducing drug (Offenburger et al., 2018).

Thioredoxin system proteins are key players in many important cellular processes, including the maintenance of redox homeostasis (Arnér, 2009; Lu and Holmgren, 2014). Regarding the influence of thioredoxins in the cellular response to cisplatin, in mammalian cells it has been shown that a delicate balance exists between the cytotoxic effect of the direct interaction with cisplatin and the protective effect, maintaining the redox equilibrium (Anestål et al., 2008; Cebula et al., 2015). Studies in cell lines suggest that cisplatin cytotoxicity is promoted by a direct interaction between cisplatin and selenocysteine amino acids, leading to the consequent formation of SecTRAPs (Anestål et al., 2008). We confirmed the cisplatin-induced cytotoxicity of TRXR-1 and, generating two distinct missense mutations by CRISPR/Cas9, we demonstrated that this cytotoxicity relies on the presence of a single selenocystein (Sec) at the C-terminal of the protein. Interestingly, the presence of Cys instead of Sec in TRXR1 can be modulated by the presence of the micronutrient selenium in the diet of rats (Lu et al., 2009).

The multifactorial nature of cisplatin resistance hampers the finding of a unique solution to overcome the resistance of tumors to cisplatin. However, there are many parallels between the response of *C. elegans* and mammals to a cisplatin treatment that would facilitate the study in nematodes of particular genetic, metabolic and environmental factors influencing the resistance to cisplatin. This information would be of great help to identify predictive biomarkers and investigate new drugs to have more effective and personalized cisplatin-based therapies.

## MATERIALS AND METHODS

### Nematode strains and general methods

*Caenorhabditis elegans* strains were cultured and maintained using standard procedures (Porta-de-la-Riva et al., 2012; Stiernagle, 2006). The N2 (Bristol) strain was used as wild-type in all experiments, and the following alleles and transgenic strains were used in this study: BS553, *fog-2(oz40) V*; XF132, *polh-1(if31) III*; CB1370, *daf-2(e1370) III*; TJ1, *cep-1(gk138) I*; CE1255, *cep-1(ep347) I*; FX536, *ced-13(tm536) X*; MD792, *ced-13(sv32) X*; VB1414, *trxr-1(sv47) IV*; CER170, *trxr-1(cer4*[U666C]*)* *IV*; CER171, *trxr-1(cer5*[U666stop]*)* *IV*; CF1038, *daf-16(mu86) I*; TJ356, *zIs356 [daf-16p::daf-16a/b::GFP + rol-6(su1006)] IV*; WS1973, *opIs56 [egl-1p::2xNLS::GFP]*; CER192, *cep-1(gk138) I, opIs56(egl-1p::GFP)*; and CL2166, *dvIs19 [pAF15(gst-4p::GFP::NLS)] III*.

### CRISPR/Cas9

To generate the *trxr-1(cer4*[U666C]*)* and *trxr-1(cer5*[U666Stop]*)* point mutation alleles, we targeted a site near the selenocysteine codon with CRISPR/Cas9. To make the sgRNA expression plasmid, we cloned the annealed oligonucleotides into *Bsa*I-digested U6::sgRNA pMB70 (Waaijers et al., 2013). Repair templates containing desired modifications were designed by Gibson Assembly Cloning Kit (New England BioLabs) and cloned into the pBSK plasmid (Addgene). Oligonucleotide sequences used to generate the 5′ and 3′ 1.5 kb overlapping fragments are available on request. Both sgRNA and repair template were verified by sequencing using T4 and M13 primers, respectively. Young adult wild-type animals were injected with 30 ng/μl of sgRNA, 100 ng/μl of repair template, 30 ng/μl of *Peft-3*::Cas9 vector (Friedland et al., 2013) and 2.5 ng/μl of P*myo-2::tdTomato*. Single fluorescent progeny were isolated and screened for the presence of the mutation by PCR. We finally established and validated homozygous mutant lines by PCR and sequencing.

### RNA-sequencing analyses

A mixed population of worms representing all stages and grown in control conditions was exposed to 60 µg/ml of cisplatin. After 24 h at 20°C, cisplatin-treated worms and untreated control animals were washed with M9 buffer to remove bacteria, and total RNA was extracted using the TRIzol method. Total mRNA was subsequently enriched using the mirVana miRNA isolation kit (Ambion) followed by poly-A capture. Library construction and Illumina's HiSeq 2000 technology sequencing was performed following the manufacturer's instructions. Approximately 12 million reads (100 bp length) for each sample were processed and aligned using TopHat software to the *C. elegans* reference genome, version WBcel235.74, to produce BAM files [Gene Expression Omnibus (GEO) database reference GSE111654]. These BAM files were analyzed with the SeqSolve NGS software (Integromics, S.L.), using a false discovery rate of 0.05 and filtering reads displaying multiple mapping sites. SeqSolve uses Cufflinks and Cuffdiff (Trapnell et al., 2012) programs to perform differential gene expression analyses between samples (*P*<0.005). Expression values were normalized in FPKM (fragments per kilobase of exon per million fragments mapped). Datasets of up- and downregulated genes were generated, comparing the differential gene expression analyses of two independent replicates (*P*<0.01 and *P*<0.05).

### Cisplatin assay during larval development

A synchronized population of L1-arrested larvae was cultured on NGM plates containing fresh OP50 and 0-200 µg/ml of cisplatin (Sigma). The body length of ≥50 worms for each condition was measured at 48, 72 and 96 h at 20°C at the stereomicroscope using NIS-Elements 3.2 imaging system. The subsequent analyses were performed with 60 µg/ml of cisplatin in the same conditions except for assays containing *daf-2(e1370)* strain, which were performed at 15°C, measuring the body length after 4 days of incubation to avoid temperature-related developmental delay. Each assay was done in duplicate, and at least two biological replicates were performed. Nonparametric Student's *t*-test (GraphPad Prism 5) was used to determine the significance of differences in the mean.

### Analysis of the effect of cisplatin in the germline

To analyze the effect of cisplatin on the germline, a synchronized population of L4-stage animals grown in standard conditions (39 h at 20°C) was transferred onto NGM plates containing 60 µg/ml cisplatin. After 24 h of incubation at 20°C, worms were washed in PBS and germlines dissected in worms anesthetized with 3 mM of levamisol in PBS, fixed with 4% paraformaldehyde and stained with DAPI (0.6 µg/ml). These stained gonads were photographed using a Nikon ECLIPSE TI-s inverted microscope. For germ cell quantification in the proliferative region, the total number of cells in a single *z*-stack within 50 µm of the distal end of the gonad was counted. At least 15 germlines were counted for each experiment.

### Brood size and unfertilized oocytes

To perform the self-fertilization assays, a synchronized population of L3-stage worms was exposed to NGM plates, with addition of fresh OP50 and 0, 60 or 100 µg/ml of cisplatin for 24 h. A total of 12 worms from each condition were selected onto fresh OP50 plates, counting the progeny and the number of oocytes laid after 3 days. A similar protocol was performed for crossing assays, where we used either a male-enriched population in the case of N2, or the male–female population of the *fog-2(oz40)* allele. After 24 h of exposure to cisplatin, a total of 12 genetic crosses were performed for each condition, placing one hermaphrodite, or female in the case of *fog-2(oz40)*, and four males on fresh OP50 plates, letting them mate and lay progeny for 3 days. This experiment was performed in duplicate. A nonparametric Student's *t*-test was used to determine the significance of differences in the mean.

### *In vivo* intracellular localization of DAF-16

To determine the subcellular localization of DAF-16, a synchronized L2-stage population of worms carrying the DAF-16::GFP transgene (TJ356) was transferred to M9 buffer containing 0, 90 or 180 µg/ml of cisplatin for 5 h at 20°C. Then, the worms were washed in M9 and mounted on a microscope slide containing a 2% agar pad, using a drop of 3 mM levamisole to anesthetize them. We quantified the DAF-16 subcellular localization by considering as ‘nuclear’ worms showing a mainly nuclear GFP accumulation along the whole body, as shown in Fig. 3B. A total of 50 worms were observed in each one of three independent replicates.

### *egl*-p::GFP expression assays

To determine ectopic *egl-1* expression, a synchronized L1-stage population of *egl-1*p::GFP (WS1973) transgenic nematodes was grown on NGM plates containing fresh OP50 and 60 μg/ml cisplatin plates for 24 h at 20°C. For *in vivo* observation, worms were recovered with M9 buffer, washed and mounted on a microscope slide containing a 2% agar pad, using a drop of 3 mM levamisole to anesthetize them. We used a Nikon ECLIPSE TI-s inverted microscope to quantify the number of cells showing an ectopic GFP signal. These experiments were performed three independent times.

### RNAi and *in vivo gst-4* expression assay

*daf-16* and *skn-1* RNAi clones used in this study were obtained from the ORFeome library (Rual et al., 2004). RNAi by feeding was performed in standard conditions, using NGM plates supplemented with 50 μg/μl ampicillin, 12.5 μg/μl tetracycline and 3 mM IPTG. To analyze *gst-4* induction, a synchronized L1-stage population of *gst-4*p::GFP reporter strain was grown on plates containing the corresponding RNAi clone for 24 h at 20°C. Then, worms were transferred to new RNAi plates including 0 or 60 μg/ml cisplatin for 24 h. Then, worms were recovered with M9 buffer, washed and mounted on a microscope slide containing a 2% agar pad, using a drop of 3 mM levamisole to anesthetize them. A Nikon ECLIPSE TI-s inverted microscope was used for *in vivo* observation, and image analysis was performed using ImageJ software. Average pixel intensity was calculated by sampling 30 worms in each assay.

### Southwestern blot analysis for Pt–DNA adducts

The southwestern blot analysis was performed using DNA extracted from a synchronized population of *C. elegans* after 24 h incubation with or without cisplatin. Genomic DNA (0.5 µg each sample) from *C. elegans* and from NRK-52E rat cells (positive control) was isolated using the DNeasy Blood and Tissue kit (Qiagen), denatured by heating (10 min, 95°C) and cooled on ice. After adding 100 µl ice-cold ammonium acetate (2 M), the DNA was transferred onto a nitrocellulose membrane, which was previously soaked in 1 M ammonium acetate. After washing (1 M ammonium acetate and water), the membrane was baked for 2 h at 80°C before being blocked in 5% non-fat milk in TBS/0.1% Tween 20 overnight at 4°C. Incubation with the primary antibody directed against Pt–DNA adducts (1:2000; Abcam; ab103261) was conducted for 1 h at room temperature. Visualization of the antibody signal was done by chemiluminescence (Bio-Rad ChemiDoc Touch Imaging System). Additionally, the membrane was stained with Methylene Blue (MP Biomedicals) to ensure equal DNA loading.

### Genetic interaction with *gsr-1* assay

This procedure was performed as previously described (Stenvall et al., 2011). Five L4 worms of the corresponding genotype were transferred to plates containing *gsr-1(RNAi)* bacteria for 24 h at 20°C. Then worms were transferred to new fresh *gsr-1(RNAi)* plates, allowed to lay eggs for 12 h and then removed. After 3 days at 20°C, larval arrest was analyzed only on day 2 plates.

## Supplementary Material

Supplementary information

First Person interview

## References

[DMM033506C1] Amable L. (2016). Cisplatin resistance and opportunities for precision medicine. *Pharmacol. Res.* 106, 27-36. 10.1016/j.phrs.2016.01.001 26804248

[DMM033506C2] Anestål K., Prast-Nielsen S., Cenas N. and Arnér E. S. J. (2008). Cell death by SecTRAPs: Thioredoxin reductase as a prooxidant killer of cells. *PLoS ONE* 3, e1846 10.1371/journal.pone.0001846 18382651 PMC2268967

[DMM033506C3] Arnér E. S. J. (2009). Focus on mammalian thioredoxin reductases—Important selenoproteins with versatile functions. *Biochim. Biophys. Acta* 1790, 495-526. 10.1016/j.bbagen.2009.01.014 19364476

[DMM033506C4] Barsyte D., Lovejoy D. A. and Lithgow G. J. (2001). Longevity and heavy metal resistance in daf-2 and age-1 long-lived mutants of Caenorhabditis elegans. *FASEB J.* 15, 627-634. 10.1096/fj.99-0966com 11259381

[DMM033506C5] Blackwell T. K., Steinbaugh M. J., Hourihan J. M., Ewald C. Y. and Isik M. (2015). SKN-1/Nrf, stress responses, and aging in Caenorhabditis elegans. *Free Radic. Biol. Med.* 88, 290-301. 10.1016/j.freeradbiomed.2015.06.008 26232625 PMC4809198

[DMM033506C6] Boyd W. A., Crocker T. L., Rodriguez A. M., Leung M. C. K., Wade Lehmann D., Freedman J. H., Van Houten B. and Meyer J. N. (2010). Nucleotide excision repair genes are expressed at low levels and are not detectably inducible in Caenorhabditis elegans somatic tissues, but their function is required for normal adult life after UVC exposure. *Mutat. Res.* 683, 57-67. 10.1016/j.mrfmmm.2009.10.008 19879883 PMC2799044

[DMM033506C7] Bujan L., Walschaerts M., Moinard N., Hennebicq S., Saias J., Brugnon F., Auger J., Berthaut I., Szerman E., Daudin M. et al. (2013). Impact of chemotherapy and radiotherapy for testicular germ cell tumors on spermatogenesis and sperm DNA: a multicenter prospective study from the CECOS network. *Fertil. Steril.* 100, 673-680.e2. 10.1016/j.fertnstert.2013.05.018 23755953

[DMM033506C8] Cebula M., Schmidt E. E. and Arnér E. S. J. (2015). TrxR1 as a potent regulator of the Nrf2-Keap1 response system. *Antioxid Redox Signal.* 23, 823-853. 10.1089/ars.2015.6378 26058897 PMC4589110

[DMM033506C9] Chang E. M., Lim E., Yoon S., Jeong K., Bae S., Lee D. R., Yoon T. K. and Choi Y. (2015). Cisplatin induces overactivation of the dormant primordial follicle through PTEN/AKT/FOXO3α pathway which leads to loss of ovarian reserve in mice. *PLoS ONE* 10, 1-16. 10.1371/journal.pone.0144245 PMC4699462 26656301

[DMM033506C10] Collis S. J., Barber L. J., Ward J. D., Martin J. S. and Boulton S. J. (2006). C. elegans FANCD2 responds to replication stress and functions in interstrand cross-link repair. *DNA Repair* 5, 1398-1406. 10.1016/j.dnarep.2006.06.010 16914393

[DMM033506C12] Dasari S. and Bernard Tchounwou P., (2014). Cisplatin in cancer therapy: Molecular mechanisms of action. *Eur. J. Pharmacol.* 740, 364-378. 10.1016/j.ejphar.2014.07.025 25058905 PMC4146684

[DMM033506C13] Dilruba S. and Kalayda G. V. (2016). Platinum-based drugs: past, present and future. *Cancer Chemother. Pharmacol.* 77, 1103-1124. 10.1007/s00280-016-2976-z 26886018

[DMM033506C15] Ellis R. and Schedl T. (2007). Sex determination in the germ line. *WormBook* (ed. The *C. elegans* Research Community). WormBook, doi/10.1895/wormbook.1.82.2. 10.1895/wormbook.1.82.2 PMC4781493 18050498

[DMM033506C16] Ermolaeva M. A. and Schumacher B. (2013). The innate immune system as mediator of systemic DNA damage responses. *Commun. Integr. Biol.* 6, e26926 10.4161/cib.26926 25210580 PMC3922787

[DMM033506C17] Fernandez De Mattos S., Clardy J. and Lam E. W.-F. (2008). FOXO3a mediates the cytotoxic effects of cisplatin in colon cancer cells. *Mol. Cancer Ther.* 7, 3237-3246. 10.1158/1535-7163.MCT-08-0398 18852127 PMC2748241

[DMM033506C18] Friedland A. E., Tzur Y. B., Esvelt K. M., Colaiácovo M. P., Church G. M. and Calarco J. A. (2013). Heritable genome editing in C. elegans via a CRISPR-Cas9 system. *Nat. Methods* 10, 741-743. 10.1038/nmeth.2532 23817069 PMC3822328

[DMM033506C19] Galanski M. (2006). Recent developments in the field of anticancer platinum complexes. *Recent Patents Anticancer Drug Discov.* 1, 285-295. 10.2174/157489206777442287 18221042

[DMM033506C20] Galluzzi L., Vitale I., Michels J., Brenner C., Szabadkai G., Harel-Bellan A., Castedo M. and Kroemer G. (2014). Systems biology of cisplatin resistance: past, present and future. *Cell Death Dis.* 5, e1257 10.1038/cddis.2013.428 24874729 PMC4047912

[DMM033506C21] Gañán-Gómez I., Wei Y., Yang H., Boyano-Adánez M.-C. and Garc, í-Manero G. (2013). Oncogenic functions of the transcription factor Nrf2. *Free Radic. Biol. Med.* 65, 750-764. 10.1016/j.freeradbiomed.2013.06.041 23820265

[DMM033506C22] Gartner A., Milstein S., Ahmed S., Hodgkin J. and Hengartner M. O. (2000). A conserved checkpoint pathway mediates DNA damage--induced apoptosis and cell cycle arrest in C. elegans. *Mol. Cell* 5, 435-443. 10.1016/S1097-2765(00)80438-4 10882129

[DMM033506C23] Gartner A., Macqueen A. J. and Villeneuve A. M. (2004). Methods for analyzing checkpoint responses in Caenorhabditis elegans. *Methods Mol. Biol.* 280, 257-274. 15187259 10.1385/1-59259-788-2:257

[DMM033506C24] Gonzalez-Exposito R., Merino M. and Aguayo C. (2016). Molecular biology of testicular germ cell tumors. *Clin. Transl. Oncol.* 18, 550-556. 10.1385/1-59259-788-2:257 26482724

[DMM033506C25] Greiss S., Schumacher B., Grandien K., Rothblatt J. and Gartner A. (2008). Transcriptional profiling in C. elegans suggests DNA damage dependent apoptosis as an ancient function of the p53 family. *BMC Genomics* 9, 334 10.1186/1471-2164-9-334 18627611 PMC2491638

[DMM033506C26] Hayden A., Douglas J., Sommerlad M., Andrews L., Gould K., Hussain S., Thomas G. J., Packham G. and Crabb S. J. (2014). The Nrf2 transcription factor contributes to resistance to cisplatin in bladder cancer. *Urol. Oncol.* 32, 806-814. 10.1016/j.urolonc.2014.02.006 24837013

[DMM033506C27] Hemmingsson O. Kao G., Still M. and Naredi P. (2010). ASNA-1 activity modulates sensitivity to cisplatin. *Cancer Res.* 70, 10321-10328. 10.1158/0008-5472.CAN-10-1548 20966125

[DMM033506C28] Honda Y. and Honda S. (1999). The daf-2 gene network for longevity regulates oxidative stress resistance and Mn-superoxide dismutase gene expression in Caenorhabditis elegans. *FASEB J.* 13, 1385-1393. 10.1096/fasebj.13.11.1385 10428762

[DMM033506C29] Honnen S. (2017). Caenorhabditis elegans as a powerful alternative model organism to promote research in genetic toxicology and biomedicine. *Arch. Toxicol.* 91, 2029-2044. 10.1007/s00204-017-1944-7 28299394

[DMM033506C30] Hou X., Bai X., Gou X., Zeng H., Xia C., Zhuang W., Chen X., Zhao Z., Huang M. and Jin J. (2015). 3′,4′,5′,5,7-pentamethoxyflavone sensitizes Cisplatin-resistant A549 cells to Cisplatin by inhibition of Nrf2 pathway. *Mol. Cells* 38, 396-401. 10.14348/molcells.2015.2183 25843086 PMC4443280

[DMM033506C31] Huang D. W., Sherman B. T. and Lempicki R. A. (2009). Systematic and integrative analysis of large gene lists using DAVID bioinformatics resources. *Nat. Protoc.* 4, 44-57. 10.1038/nprot.2008.211 19131956

[DMM033506C32] Kadandale P. and Singson A. (2004). Oocyte production and sperm utilization patterns in semi-fertile strains of Caenorhabditis elegans. *BMC Dev. Biol.* 4, 3 10.1186/1471-213X-4-3 15086962 PMC404374

[DMM033506C33] Kaletta T. and Hengartner M. O., (2006). Finding function in novel targets: C. elegans as a model organism. *Nat. Rev. Drug Discov.* 5, 387-398. 10.1038/nrd2031 16672925

[DMM033506C34] Kaspar J. W., Niture S. K. and Jaiswal A. K. (2009). Nrf2:INrf2 (Keap1) signaling in oxidative stress. *Free Radic. Biol. Med.* 47, 1304-1309. 10.1016/j.freeradbiomed.2009.07.035 19666107 PMC2763938

[DMM033506C35] Kelland L. (2007). The resurgence of platinum-based cancer chemotherapy. *Nat. Rev. Cancer* 7, 573-584. 10.1038/nrc2167 17625587

[DMM033506C36] Krüger K., Ziegler V., Hartmann C., Henninger C., Thomale J., Schupp N. and Fritz G. (2016). Lovastatin prevents cisplatin-induced activation of pro-apoptotic DNA damage response (DDR) of renal tubular epithelial cells. *Toxicol. Appl. Pharmacol.* 292, 103-114. 10.1016/j.taap.2015.12.023 26739623

[DMM033506C38] Lettre G. and Hengartner M. O. (2006). Developmental apoptosis in C. elegans: a complex CEDnario. *Nat. Rev. Mol. Cell Biol.* 7, 97-108. 10.1038/nrm1836 16493416

[DMM033506C39] Li W., Bandyopadhyay J., Hwaang H. S., Park B.-J., Cho J. H., Lee J. I., Ahnn J. and Lee S.-K. (2012). Two thioredoxin reductases, trxr-1 and trxr-2, have differential physiological roles in Caenorhabditis elegans. *Mol. Cells* 34, 209-218. 10.1007/s10059-012-0155-6 22836943 PMC3887811

[DMM033506C40] Lu J., Zhong L., Lönn M. E., Burk R. F., Hill K. E. and Holmgren A. (2009). Penultimate selenocysteine residue replaced by cysteine in thioredoxin reductase from selenium-deficient rat liver. *FASEB J.* 23, 2394-2402. 10.1096/fj.08-127662 19351701 PMC2717770

[DMM033506C41] Lu J. and Holmgren A. (2014). The Thioredoxin Superfamily in Oxidative Protein Folding. *Antioxid Redox Signal.* 21, 457-470. 10.1089/ars.2014.5849 24483600

[DMM033506C43] Meier B., Cooke S. L., Weiss J., Bailly A. P., Alexandrov L. B., Marshall J., Raine K., Maddison M., Anderson E., Stratton M. R. et al. (2014). C. elegans whole genome sequencing reveals mutational signatures related to carcinogens and DNA repair deficiency. *Genome Res.* 24, 1624-1636. 10.1101/gr.175547.114 25030888 PMC4199376

[DMM033506C44] Murakami S. and Johnson T. E. (1996). A genetic pathway conferring life extension and resistance to UV stress in Caenorhabditis elegans. *Genetics* 143, 1207-1218. 8807294 10.1093/genetics/143.3.1207 PMC1207391

[DMM033506C45] Murphy C. T. and Hu P. J. (2013). Insulin/insulin-like growth factor signaling in C. elegans. *WormBook* 1-43. 10.1895/wormbook.1.164.1 PMC4780952 24395814

[DMM033506C48] Nehme R. and Conradt B. (2008). egl-1: a key activator of apoptotic cell death in C. elegans. *Oncogene* 27, S30-S40. 10.1038/onc.2009.41 19641505

[DMM033506C49] Nijman S. M. B. (2015). Functional genomics to uncover drug mechanism of action. *Nat. Chem. Biol.* 11, 942-948. 10.1038/nchembio.1963 26575241

[DMM033506C50] Offenburger S.-L., Jongsma E. and Gartner A. (2018). Mutations in Caenorhabditis elegans neuroligin-like glit-1, the apoptosis pathway and the calcium chaperone crt-1 increase dopaminergic neurodegeneration after 6-OHDA treatment. K. A. Caldwell, ed. *PLoS Genet.* 14, e1007106. 29346364 10.1371/journal.pgen.1007106 PMC5773152

[DMM033506C51] Oliveira R. P., Abate J. P., Dilks K., Landis J., Ashraf J., Murphy C. T. and Blackwell T. K. (2009). Condition-adapted stress and longevity gene regulation by Caenorhabditis elegans SKN-1/Nrf. *Aging Cell* 8, 524-541. 10.1111/j.1474-9726.2009.00501.x 19575768 PMC2776707

[DMM033506C52] Ortiz M. A., Noble D., Sorokin E. P. and Kimble J. (2014). A new dataset of spermatogenic vs. oogenic transcriptomes in the nematode Caenorhabditis elegans. *G3* 4, 1765-1772. 10.1534/g3.114.012351 25060624 PMC4169169

[DMM033506C54] Park C.-M., Park M. J., Kwak H. J., Moon S. I., Yoo D. H., Lee H. C., Park I. C., Rhee C. H. and Hong S. I. (2006). Induction of p53-mediated apoptosis and recovery of chemosensitivity through p53 transduction in human glioblastoma cells by cisplatin. *Int. J. Oncol.* 28, 119-125. 10.3892/ijo.28.1.119 16327987

[DMM033506C55] Park S.-K., Tedesco P. M. and Johnson T. E. (2009). Oxidative stress and longevity in Caenorhabditis elegans as mediated by SKN-1. *Aging Cell* 8, 258-269. 10.1111/j.1474-9726.2009.00473.x 19627265 PMC2762118

[DMM033506C56] Porta-De-La-Riva M., Fontrodona L., Villanueva A. and Cerón J. (2012). Basic Caenorhabditis elegans methods: synchronization and observation. *J. Vis. Exp*. e4019 10.3791/4019 22710399 PMC3607348

[DMM033506C57] Rodriguez M. Snoek L. B., De Bono M. and Kammenga J. E. (2013). Worms under stress: C. elegans stress response and its relevance to complex human disease and aging. *Trends Genet.* 29, 367-374. 10.1016/j.tig.2013.01.010 23428113

[DMM033506C58] Roerink S. F., Koole W., Stapel L. C., Romeijn R. J. and Tijsterman M. (2012). A broad requirement for TLS polymerases η and κ, and interacting sumoylation and nuclear pore proteins, in lesion bypass during C. elegans embryogenesis. S. Jinks-Robertson, ed. *PLoS Genet.* 8, e1002800. 22761594 10.1371/journal.pgen.1002800 PMC3386174

[DMM033506C59] Roh J.-Y., Lee J. and Choi J. (2006). Assessment of stress-related gene expression in the heavy metal-exposed nematode Caenorhabditis elegans: a potential biomarker for metal-induced toxicity monitoring and environmental risk assessment. *Environ. Toxicol. Chem.* 25, 2946-2956. 10.1897/05-676R.1 17089718

[DMM033506C60] Rual J.-F., Ceron J., Koreth J., Hao T., Nicot A. S., Hirozane-Kishikawa T., Vandenhaute J., Orkin S. H., Hill D. E., van den Heuvel S. et al. (2004). Toward improving Caenorhabditis elegans phenome mapping with an ORFeome-based RNAi library. *Genome Res.* 14, 2162-2168. 10.1101/gr.2505604 15489339 PMC528933

[DMM033506C61] Schumacher B., Schertel C., Wittenburg N., Tuck S., Mitani S., Gartner A., Conradt B. and Shaham S. (2005a). *C. elegans ced-13* can promote apoptosis and is induced in response to DNA damage. *Cell Death Differ.* 12, 153-161. 10.1038/sj.cdd.4401539 15605074

[DMM033506C62] Schumacher B., Hanazawa M., Lee M.-H., Nayak S., Volkmann K., Hofmann R., Hengartner M., Schedl T. and Gartner A. (2005b). Translational repression of C. elegans p53 by GLD-1 regulates DNA damage-induced apoptosis. *Cell* 120, 357-368. 10.1016/j.cell.2004.12.009 15707894

[DMM033506C63] Siddik Z. H. (2003). Cisplatin: mode of cytotoxic action and molecular basis of resistance. *Oncogene* 22, 7265-7279. 10.1038/sj.onc.1206933 14576837

[DMM033506C64] Singh V. and Aballay A. (2009). Regulation of DAF-16-mediated innate immunity in Caenorhabditis elegans. *J. Biol. Chem.* 284, 35580-35587. 10.1074/jbc.M109.060905 19858203 PMC2790988

[DMM033506C65] Stenvall J., Fierro-Gonzalez J. C., Swoboda P., Saamarthy K., Cheng Q., Cacho-Valadez B., Arner E. S. J., Persson O. P., Miranda-Vizuete A. and Tuck S. (2011). Selenoprotein TRXR-1 and GSR-1 are essential for removal of old cuticle during molting in Caenorhabditis elegans. *Proc. Natl Acad. Sci. USA* 108, 1064-1069. 10.1073/pnas.1006328108 21199936 PMC3024696

[DMM033506C66] Stergiou L., Doukoumetzidis K., Sendoel A. and Hengartner M. O. (2007). The nucleotide excision repair pathway is required for UV-C-induced apoptosis in Caenorhabditis elegans. *Cell Death Differ.* 14, 1129-1138. 10.1038/sj.cdd.4402115 17347667

[DMM033506C67] Stiernagle T. (2006). Maintenance of C. elegans. *WormBook* (ed. The *C. elegans* Research Community). WormBook, doi/10.1895/wormbook.1.101.1. 10.1895/wormbook.1.101.1 PMC4781397 18050451

[DMM033506C69] Tatusov R. L., Fedorova N. D., Jackson J. D., Jacobs A. R., Kiryutin B., Koonin E. V., Krylov D. M., Mazumder R., Mekhedov S. L., Nikolskaya A. N. et al. (2003). The COG database: an updated version includes eukaryotes. *BMC Bioinformatics* 4, 41 10.1186/1471-2105-4-41 12969510 PMC222959

[DMM033506C70] Tawe W. N., Eschbach M.-L., Walter R. D. and Henkle-Duhrsen K. (1998). Identification of stress-responsive genes in Caenorhabditis elegans using RT-PCR differential display. *Nucleic Acids Res.* 26, 1621-1627. 10.1093/nar/26.7.1621 9512531 PMC147444

[DMM033506C71] Tepper R. G., Ashraf J., Kaletsky R., Kleemann G., Murphy C. T. and Bussemaker H. J. (2013). PQM-1 complements DAF-16 as a key transcriptional regulator of DAF-2-mediated development and longevity. *Cell* 154, 676-690. 10.1016/j.cell.2013.07.006 23911329 PMC3763726

[DMM033506C72] Trapnell C., Roberts A., Goff L., Pertea G., Kim D., Kelley D. R., Pimentel H., Salzberg S. L., Rinn J. L. and Pachter L. (2012). Differential gene and transcript expression analysis of RNA-seq experiments with TopHat and Cufflinks. *Nat. Protoc.* 7, 562-578. 10.1038/nprot.2012.016 22383036 PMC3334321

[DMM033506C73] van Haaften G., Romeijn R., Pothof J., Koole W., Mullenders L. H. F., Pastink A., Plasterk R. H. A. and Tijsterman M. (2006). Identification of conserved pathways of DNA-damage response and radiation protection by genome-wide RNAi. *Curr. Biol.* 16, 1344-1350. 10.1016/j.cub.2006.05.047 16824923

[DMM033506C74] Waaijers S., Portegijs V., Kerver J., Lemmens B. B. L. G., Tijsterman M., Van Den Heuvel S. and Boxem M. (2013). CRISPR/Cas9-Targeted Mutagenesis in *Caenorhabditis elegans*. *Genetics* 195, 1187-1191. 10.1534/genetics.113.156299 23979586 PMC3813849

[DMM033506C75] Wang D. and Lippard S. J. (2005). Cellular processing of platinum anticancer drugs. *Nat. Rev. Drug Discov.* 4, 307-320. 10.1038/nrd1691 15789122

[DMM033506C76] Yang J.-Y. and Hung M.-C. (2009). A new fork for clinical application: targeting forkhead transcription factors in cancer. *Clin. Cancer Res* 15, 752-757. 10.1158/1078-0432.CCR-08-0124 19188143 PMC2676228

[DMM033506C77] Yee C., Yang W. and Hekimi S. (2014). The intrinsic apoptosis pathway mediates the pro-longevity response to mitochondrial ROS in C. elegans. *Cell* 157, 897-909. 10.1016/j.cell.2014.02.055 24813612 PMC4454526

[DMM033506C78] Yen K., Narasimhan S. D. and Tissenbaum H. A. (2011). DAF-16/Forkhead box O transcription factor: many paths to a single Fork(head) in the road. *Antioxid. Redox Signal.* 14, 623-634. 10.1089/ars.2010.3490 20673162 PMC3021330

