# Supplementary material for: Genetic and cellular sensitivity of Caenorhabditis elegans to the chemotherapeutic agent cisplatin
Source: Dis Model Mech. 2018 Jun 21;11(6):dmm033506. doi: 10.1242/dmm.033506 (PMC6031354; doi:10.1242/dmm.033506)
Supplement: Supplementary information [file dmm-11-033506-s1.pdf]

First person – Francisco Javier García-Rodríguez

First Person is a series of interviews with the first authors of a selection of papers published in Disease Models & Mechanisms, helping early-career researchers promote themselves alongside their papers. Francisco Javier García-Rodríguez is first author on ‘Genetic and cellular sensitivity of *Caenorhabditis elegans* to the chemotherapeutic agent cisplatin’, published in DMM. Francisco conducted the research in this article while a PhD student in the lab of Julián Cerón and Alberto Villanueva at Institut d'Investigació Biomédica de Bellvitge (IDIBELL), Barcelona, Spain. He is now a postdoc at the Andalusian Centre for Developmental Biology (CABD), Seville, Spain, using *C. elegans* as a model to investigate the role of the mitochondrial prohibitin complex in aging.

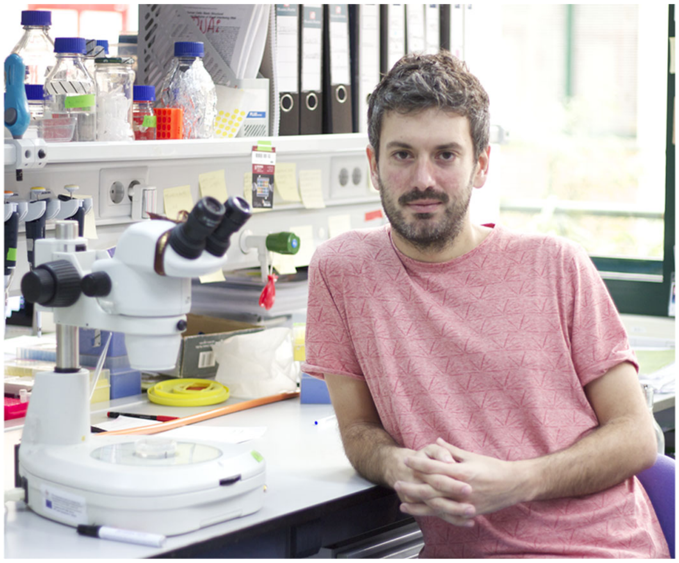

Francisco Javier García-Rodríguez

How would you explain the main findings of your paper to non-scientific family and friends?

In the field of cancer chemotherapy, cisplatin is one of the most widely prescribed drugs and an effective treatment for many cancer types, but unfortunately, some tumors are able to develop resistance to the drug. Despite the amount of information available, the cellular mechanisms behind the acquisition of cisplatin resistance remain elusive. We used *Caenorhabditis elegans*, a tiny transparent worm, as a model to better understand biological responses to cisplatin-based chemotherapy.

What are the potential implications of these results for your field of research?

In this work, we established *C. elegans* as an *in vivo* model to show that many parallels exist between the responses of worms and mammals to cisplatin. This will facilitate the use of worms as a model to study the particular genetic, metabolic and environmental factors influencing cisplatin resistance. In the future, these studies could help to identify predictive biomarkers and potential new drugs to develop more effective and personalized cisplatin-based therapies.

What are the main advantages and drawbacks of the model system you have used as it relates to the disease you are investigating?

Although worms cannot develop tumors in any of their somatic tissues, many essential genes and pathways are highly conserved between humans and worms. This makes it possible to use *C. elegans* as a model to gain insights into the molecular and cellular basis of cancer therapy. Additionally, *C. elegans* genetics allows a wide range of genetic studies, plus the generation of mutant and transgenic lines that can be used to interrogate genetic function and interactions, and perform high-throughput *in vivo* drug and genetic screenings that would be more difficult and time consuming in other model systems such as mammals.

Francisco Javier García-Rodríguez's contact details: Andalusian Centre for Developmental Biology (CABD), Universidad Pablo de Olavide, Carretera de Utrera km 1, 41013 Sevilla, Spain.
E-mail: fjgarrod1@upo.es

“What took lots of effort a few years ago by mutagenesis or bombardment, can now be accomplished in a few days by CRISPR/Cas9 techniques.”

What has surprised you the most while conducting your research?

How genome editing techniques are evolving in *C. elegans*, without any doubt. What took lots of effort a few years ago by mutagenesis or bombardment, can now be accomplished in a few days by CRISPR/Cas9 techniques. We were lucky to experience the development of this tool first hand.

Describe what you think is the most significant challenge impacting your research at this time and how will this be addressed over the next 10 years?

Cisplatin-based research advances have been notorious during the last few years. However, more work is needed to better dissect the cisplatin mechanism of action. Furthermore, I think the trend in the field of chemotherapy is leaning towards personalized medicine, and so advances in tumor characterization would allow individualized and targeted treatments. In line with this, recent advances in *C. elegans* genome editing are allowing fast and easy generation of worms that reproduce the same genetic alterations found in patients, which allows screening for better alternative individualized therapies. We like to call them ‘avatar worms’.

What changes do you think could improve the professional lives of early-career scientists?

I consider myself lucky because during this period I had the most important two things that, in my opinion, are essential for a young researcher: funding and the support of an involved and accessible

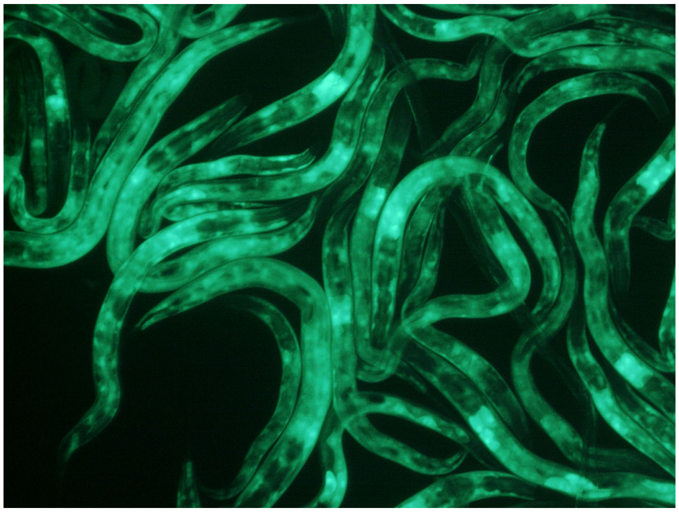

Transgenic worms carrying GFP-tagged transcriptional reporter Pgst-4 show a positive response when exposed to cisplatin.

principal investigator. But, unfortunately, funding is not always accessible here in Spain, where investment in science is decreasing year after year. Research groups have been forced to disband, or PhD students find that they need to leave their research careers and start, maybe, a promising career as a waiter, which is, in fact, better paid…

What's next for you?

After my PhD, I moved to the Andalusian Centre for Developmental Biology (CABD) in my hometown, Seville, as a postdoctoral researcher where we use *C. elegans* to get new insights into how the mitochondrial prohibitin complex affects aging.

Reference

García-Rodríguez, F. J., Martínez-Fernández, C., Brena, D., Kukhtar, D., Serrat, X., Nadal, E., Boxem, M., Honnen, S., Miranda-Vizuete, A., Villanueva, A. and Cerón, J. (2018). Genetic and cellular sensitivity of *Caenorhabditis elegans* to the chemotherapeutic agent cisplatin. *Dis. Model. Mech.* **11**: dmm033506.

Disease Models & Mechanisms
